# Socialism Order of Worth and Analytical Adequacy Axiom

**DOI:** 10.1007/s10746-022-09629-3

**Published:** 2022-06-15

**Authors:** Christian Schneijderberg

**Affiliations:** International Center for Higher Education Research (INCHER), University of Kassel, Moenchebergstrasse 17, 34109 Kassel, Germany

**Keywords:** Axiological Sociology, Orders of worth, Public and private health insurance, Sociology of conventions, Theory development

## Abstract

Boltanski and Thévenot (On justification. Economies of worth, Princeton University Press, Princeton, 2006) constructed in their seminal work *On Justification* the Orders of Worth (OW) framework as a research program for further empirical and theoretical development. This article suggests two methodological additions to extend the analytical capacities of the OW framework: The Socialism OW and the analytical adequacy axiom. The polito-philosophical Socialism OW, which acknowledges '(collective) welfare' as its *mode of evaluation (worth)* and the higher principle of 'solidarity' as its *test*, is rooted in the political philosophy of Rosanvallon (The society of equals, Harvard University Press, Cambridge, 2013). In addition to the systematic justification of the canonical text, this article also offers a tabular presentation for the construction of a new OW in relation to the axioms. In the article, first, the existing OW are put under scrutiny discussing the test category of solidarity, which was added and creates an analytic overload for the Civic OW. Second, analyzing the case of the German binary statutory health system, comprising of a private (first-class) and a public (second-class) healthcare, the capacities of the existing OW are discussed to identify a blank spot in the OW framework for empirical analysis. Accordingly, the descriptive analysis of the German binary health system is less about how the system is justified, and much more about understanding how given OW operate within it as coordinative devices. This systematic analysis of a situation of (temporary) agreements, especially of investments in forms, amends the OW use for empirical analysis of critique and justification in a situation.

## Introduction

In a situation of critique and justification (interaction dimension), reflective individuals refer to value principles (structural dimension), and, by doing so, reconstruct or modify social norms (Heinich, 2020a). Studying implicit values aims at an understanding of actors’ motivations, emotional commitment, thinking and embeddedness in formal structures and informal organizing of society. Dissimilar to the inductive methodological stance of discovering epistemes (systems of thought) in discursive formations (knowledge), Heinich (2020b) suggests a deductive methodic^1^-analytical approach of applying axiological, i.e., general philosophical values for sociological study in a specific socio-cultural context. Heinich (2020a) highlights the great potential for (axiological) sociology rooted in the analytical framework of the orders of worth (OW) developed by Boltanski and Thévenot (2006 [1991]) with others (Boltanski & Chiapello, 2007; Thévenot et al., 2000). Currently, seven OW (Civic, Domestic, Fame, Industrial, Inspired, Market, and Project or Networked; an empirically constructed nucleus of a green OW is awaiting theoretical foundations) exist.^2^ The Green OW nucleus appears to be a reminder of the OW framework not being complete, but an ongoing work-in-progress (Boltanski, 2012; Wagner, 1999).

In addition to extending the analytical use of the OW framework, the OW categories and key terms also became subject to modifications. When Thévenot et al., (2000: 241) amended the Civic OW with the words ‘welfare’ and ‘solidarity,’ they revealed, perhaps unintentionally, an incongruity in their framework. Although legal ‘equality’ can be set by establishing equal ‘rules and regulations’ and giving ‘citizens’ ‘fundamental rights,’ ‘solidarity’ cannot be set in the same way (Boltanski & Thévenot, 2006: 107–117).^3^ For example, the Civic OW addresses all aspects of the political organization of the Western liberal nation state, such as the legal organization of the Market and the constitution of the welfare state, but cannot legally organize ‘solidarity,’ which is the basis of welfare. According to Rosanvallon (2013), ‘solidarity’ derives from a sense of communality and reciprocity. Communal and reciprocal ‘solidarity’ among individuals or singularities is considered fundamental for the new Socialism OW, which is introduced in this paper. The term Socialism was selected for the new OW to emphasize the element of a sharing ‘solidarity’ among humans, which emphasizes a social institution different from the state (Vincent, 2010: 83). In spite of the chequered past of Socialism (Piketty, 2021; Vincent, 2010), Socialism emphasizes the doing well of the whole community. For example, doing well is not limited to the ‘rules and regulations’ of the capitalist welfare state (Civic, Industrial and Market OW; see also Batifoulier et al., 2021; Chiapello & Knoll, 2020; Esping-Anderson, 1990). Amending the OW framework in the spirit of the OW research program, the Socialism OW employs ‘solidarity’ as the *test* of the higher common good named ‘(collective) welfare,’ which is a value-principle in a “strictly sociological definition of value as worth” (Heinich, 2020a: 218)–i.e., extending the OW framework’s applicability for general sociological use.

The analytic overload of the Civic OW and analytical blank spot in the OW framework for general sociological analysis became obvious when analyzing the German statutory health system. One of a kind worldwide,^4^ the German basic health insurance system divides individuals in privately insured first-class (about 11% of the population; majority: males) and second-class publicly insured ‘citizens’ (Civic OW *subjects*).^5^ In the current form, the OW framework can only grasp how Market organized ‘cost’ containment initiatives have failed to supply legally secured ‘equality’ to basic health ‘infrastructures’ (Industrial OW *objects*). In the ‘collective interest’ (Civic OW *mode of evaluation (worth)*), a minimum annual income or the status as self-employed entrepreneur constitute the right to be granted private insurance, which secures better health treatment, potentially a longer, but surely a better quality of life–not just in case of falling ill. In general, the existence of such a political economy^6^ of health is a challenge for the Civic OW *test* of ‘equality.’

To exceed the German differentiation of first-class (rich(er) part of German society) and second-class (publicly health insured) ‘citizens,’ i.e., to include analytically all structural elements of a solidaristic statutory health insurance system, I introduce in the following the Socialism OW. The descriptive analysis of the German binary health system is less about how the system is justified, and much more about understanding how given OW operate within it as coordinative devices. In addition, the German statutory health system provides for the context of discovery (i.e., to discussing an analytical blank spot in the OW framework), the German statutory health system is the empirical example paving the way for justifying the introduction of the Socialism OW. Accordingly, in this paper, the empirical elaboration appears more prominent than the presentation of the theoretical basis of the Socialism OW provided by Rosanvallon (2013). I develop the Socialism OW categories based on the grammar used for the empirical analysis. The methodic-theoretical reflection of OW construction led to the formulation of the analytical adequacy axiom (a7). After the definition of the categories of the new OW, the article ends with the justification of the selected canonical text by Rosanvallon (2013) for the Socialism OW, and the explanation of Socialism OW according to the six framework axioms (Boltanski, 2012; Boltanski & Thévenot, 2006).

## The Analytical Framework of Orders of Worth

### Brief Introduction to OW Framework

According to Boltanski and Thévenot (2006), the plural OW draws on the principle of common humanity and represent the “main conventions of coordination” (Thévenot, 2001: 411). The OW are part of an action regime of justice (Boltanski, 2012: 75–78). As Boltanski and Thévenot (1999: 362) write: “The regime of justification, which requires collective conventions of equivalence, is only needed when more local regimes of coordination based on either “personal convenience” or “conventional utilization” are not sufficient to deal with the misfortune of a situation and determine what is convenient or appropriate”. In situations of justification, the reflective actor is required to “recognize the nature of the situation and apply the appropriate principle[s] of justice” (Boltanski & Thévenot, 2006: 146). The analytical goal of the OW is thus to “uncover the common requirements shared by all orders and to account for a variety of modes of acting that may qualify for public legitimacy” (Thévenot et al., 2000: 239). Accordingly, the OW are both context-dependent (i.e., dependent on a specific space in time) and context-transcendent (i.e., humane references for orientation) (Susen, 2017: 358f.).

Associated with the new French *Économie des Conventions* (e.g., Diaz-Bone & Salais, 2011: 9f.; Diaz-Bone, 2018: 42–44), the analytical OW framework by Boltanski and Thévenot (2014) does account for the core commonality of the pragmatic study of institutionalized social structures as conventions.

For the E[conomie des] C[onventions], the name giving notion of convention is important. Conventions are not to be confused with arbitrary “standards” or traditional customs or ad hoc agreements. Conventions are understood as shared interpersonal logics how to coordinate and to evaluate actions, individuals and objects in situations of uncertainty […]. Conventions are socio-cultural resources for the coordination between actors. (Diaz-Bone, 2011: 46)

Differences between the OW and *Économie des Conventions* surface looking at the pragmatic use (of parts) of the OW in *Économie des Conventions* studies (e.g., Batifoulier et al., 2021; Chiapello & Knoll, 2020; Storper & Salais, 1997), which are rather different to the methodic-theoretical OW construction and application (Blokker, 2011; Boltanski, 2012; Boltanski & Thévenot, 2006; Heinich, 2020a, 2020b; Wagner, 1999). For example, Batifoulier et al., (2021: 36) state that “[t]here is no universal definition of national solidarity; there are, however, several interpretations of solidarity. These analytical grids can be described as conventions” and provide a historical “interpretative framework” for the “progressive shift from a Fordist convention to a liberal convention” (Batifoulier et al., 2021: 39, 37) of complementary, private health insurance in France.

In comparison to the broader *Économie des Conventions*, the OW presents the “first fine-grained, multi-layered and systematic sociological account of the role of justificatory practices in human life forms” (Susen, 2017: 352). The axiological, methodic-theoretical construction of a OW requires the combined effort of a polito-philosophical theoretical basis and an empirical elaboration (Boltanski, 2012: 98f.). The analytical OW framework roots in classical political philosophies epitomizing different orders (also referred to as worlds and polities): Jean-Jacques Rousseau (Civic), Jaques Bénigne Bossuet (Domestic), Thomas Hobbes (Fame), Claude-Henri de Saint-Simon (Industrial), Aurelius Augustine (Inspired) and Adam Smith (Market). The Civic OW represents the political organization of the Western liberal nation state. The conventions of the Domestic OW are those of esteemed individuals in the family context. The Fame OW focuses on the publicly known being, and the Inspired OW focuses on the creative being. The Industrial OW represents the conventions of technical organizations and institutions, whereby the Market OW is the institutionalization of Market competition. The Project or Networked OW lately amended this framework with a detailed analysis in *The New Spirit of Capitalism* (Boltanski & Chiapello, 2007).^7^ The OW framework also includes an empirical elaboration for a Green OW, still waiting for a polito-philosophical theoretical basis (Thévenot et al., 2000).

In two overview articles, Boltanski and Thévenot (1999, 2000) present the categories of an analytical framework, which supplies core codes or terms to identify each OW. These categories are called *mode of evaluation (worth)* (which denotes the impersonal and, therefore, higher principle of each OW (Boltanski & Thévenot, 1999: 361), *test*, *form of relevant proof*, *qualified objects*, *qualified human beings*, *time formation*, and *space formation* (Thévenot et al., 2000: 241). These categories can guide sociological analysis while responding to specific meaning questions: *mode of evaluation (worth)* (how-question), *test* (why-question), *form of relevant proof* (whereby-question), *qualified subjects* (who-question), *qualified objects* (what-question), *space formation* (where-question) and *time formation* (when-question). In the section below, Table 1 illustrates this explanation of the OW framework and shows/presents the categories of the Civic, Industrial and Market OW.

### Micro-, Meso- and Maco-Levels of Conventions Situatedness: Justification and Critique *in* a Situation and *of* a Situation as it is

In spite of the association to the foundational economic approach named the *Économie des Conventions* (EC), the economic actors are just one kind among the many social actors captured by the plural, sociological OW framework by Boltanski and Thévenot (2006). Actors can be individuals (sociological micro-level), collectives and organizations (meso-level) as well as nation states (macro-level). In an abstract manner, Thévenot (2000) defines non-individual actors as forms, which are constructed by all kinds of investments. Investments are not just economic investments that can be counted but all elements that count in a situation of conflict and justification. As a “generalization of coordination” the “investment in form” (e.g., a national statutory health and education system) “is costly and demands negotiation and material equipment, but the cost may be offset by returns in coordination which depend on the extension of the domain of time and space within which it is accepted” (Thévenot, 2001: 407).

To study the situatedness of critique and justification of forms, Schneijderberg (2020: 107) extends the notion of presence (in a situation) towards the past by theoretically elaborating on “justification *by* a situation *as it is*”. Studying the valorization of performances of universities (Industrial OW *test* of ‘(technical) efficiency’), Schneijderberg (2020) shows how universities fence off critique and justify public expenditure by using indicators presenting (excellent) past academic performances (e.g., third-party funding), which are simultaneously geared towards the future investment in the form of a university. Similar to the situation transcending OW conventions for coordination, the Industrial OW indicator-based justification is a form of agreement focusing on a “convention of equivalence external to themselves” (Boltanski & Thévenot, 1999: 361). Schneijderberg (2020) presents a viable empirical solution on how to address agreements for forms to avoid permanent situations of conflict, which as subject to recurrent Industrial OW evaluations and valorization of university performances.

For methodological OW framework development, in the following, the present state of the German binary health system is analyzed in what will appear to the (OW sympathetic) reader as a rather unduly static manner. The static manner is undeniably rooted in the systematic, theoretic-methodic and criteria-led extension of the OW framework. As a critique *of* a situation *as it is*–both of the OW and the German binary health system–this paper constructs a situation ending with the elaboration of the Socialism OW and analytical adequacy axiom. Accordingly, for methodological OW framework development the social processes of justification, critique, tests or other forms of coordination play no significant role. Instead, OWs are used as dimensions detectable within the fabric of the German binary healthcare system to understand how given OW operate within it as coordinative devices and empirically justify the Socialism OW.

## Discussion of an Analytical Blank Spot in the OW Framework

### Methodological Problem Discovery in OW Framework

In a first attempt to include health and other social welfare benefits, such as pension, subsistence and unemployment schemes, Thévenot et al., (2000: 241) changed the definition of Civic OW *mode of evaluation (worth)* from “collective interest” (Boltanski & Thévenot, 1999: 368) to “collective welfare”. Additionally, Thévenot et al., (2000: 241) combined ‘solidarity’ (originally used in the *elementary relation* category) and ‘equality’ (originally in *human qualification*) in the ‘test’ category, while also replacing the category of ‘human qualification’ with that of *qualified human beings*, which they defined as ‘equal citizens’ and ‘solidarity unions’. Thévenot et al., (2000: 246) write that “[j]ustifications based on the Civic equality or solidarity refer to collective welfare as the standard of evaluation, and proposes or opposes projects [based] on such goals as equal access and protection of civil rights”. However, the combination of equality and solidarity constructs both a theoretical-analytical and social order problem. Especially Alexander (1997: 115)^8^ stresses: “The solidarity sphere, in principle and in practice, can be differentiated not only from markets and states but from such other noncivil spheres as religion, family and science”. Alexander (1997: 115) adds, “solidarity and social values” influence “what and how we speak, think and feel about politics”. Accordingly, solidarity has a theoretically and empirically different quality than equality. This suggests that ‘solidarity’ can become part of the political struggle for ‘equality–and equality-oriented conflict addressing the absence and/or justifying ‘solidarity’ as a higher common good, i.e., the *test* category contributing to the *mode of evaluation (worth)* of an OW (Boltanski & Thévenot, 2006: 66, 125) in an action regime of justice. In addition, neither the explanations of the normative roots of the Civic OW by Boltanski and Thévenot (2006: 107–117) nor the grammar of the Civic OW (Boltanski & Thévenot, 2006: 239–318) justify the replacement of ‘collective interest’ with ‘collective welfare’ (Thévenot et al., 2000: 241).

The addition of ‘solidarity’ to the Civic OW test category by Thévenot et al. (2000) is interpreted by the author as Thévenot et al. (2000) not considering ‘solidarity’ a compromise or composite arrangement between two already existing OW. In the existing OW framework, in addition to the Civic OW, only the Domestic OW contains homeopathic traces, which could be identified as being somehow related to ‘solidarity’. According to Boltanski and Théventon (2006: 90–98), in the Domestic OW, social order of subjects depends on the hierarchy of trust and reciprocal personal dependence. However, eventual ‘solidarity’ is not a generic characteristic as it depends on a traditional, local knowing one's place in authoritarian family ties and work place settings. The specific Domestic OW qualities of important and worthy persons (e.g., Bossuets’ king) are “to be distinguished, straightforward, faithful and to have character” (Boltanski & Thévenot, 1999: 370). The Domestic OW categoric features are ‘esteem, reputation’ (*mode of evaluation (worth)*), ‘trustworthiness’ (*test*), ‘oral, exemplary, personally warranted’ (*form of relevant proof*), ‘patrimony, locale, heritage (*qualified objects*), ‘authority’ (*qualified human beings*), ‘customary past’ (*time formation*), and ‘local, proximal anchoring’ (*space formation*).

In comparison to the Domestic OW, the Civic OW “formula for subordination” and “access to worth does not depend on […] the position one occupies in a hierarchical chain of dependencies” (Boltanski & Thévenot, 2006: 107). In the Civic OW, the “civil peace and the common good […] [are] placed above private interests” (Boltanski & Thévenot, 2006: 107), which define a disembodied political worth. However, while ‘equality’ does *test* for disembodied worth, ‘solidarity’ does *test*, in particular, for embodied (health) needs (e.g., Knotz et al., 2021; Miller, 1999). Accordingly, without adding Socialism as a new OW and a principal of equivalence “the frame of analysis” cannot “tackle agreement and disagreement with the same tool” (Boltanski & Thévenot, 1999: 360) concerning ‘solidarity’ as a *test* for social order in political dispute.

### Theoretical Blank Spot in the OW Framework

Continually reflecting the idea that the Civic OW does not quite fit together and that both the *test* of ‘equality’ and ‘solidarity’ are an analytic overload of the Civic OW, I applied the OW framework as an empirical case study of the German binary health insurance system. Currently, the political economy in the binary health system is based on the Bismarckian equilibrium of acceptable socio-economic in ‘equality’ and Civic stability for the rich(er) members of German society. The display of non- ‘solidarity’ by the 11% of individuals with private health insurance illustrates an in’equality’ of human rights and a lack of esteem for fellow ‘citizens’ (Civic OW subjects).

The escape of the privately insured rich(er) and self-employed from the ‘solidaristic community’ constitutes a refusal to recognize the health and survival needs of others. Having to assert to the German binary health system the classification of first-class, rich(er) ‘citizens’ and second-class, poorer ‘citizens’ means moral imbalance of the German ‘collective interest’. According to Norton (1991: 21), this can be explained by modern morality having a meaning equal to minimal rules-obedience as it “enlists morality alongside law for the preservation of social order”. Concluding his book on democracy, moral virtue and economic liberalism, Norton (1991: 176) writes about the great costs for an individual’s life well lived (Greek: *eudaimonia*), because the acceptance of “an economistic conception of self and society that has by its moral minimalism rendered invisible the large demands and rewards of worthy living”. For example, being treated by the chief physician, which is not covered by public health insurance in the growing health market in Germany (Busse et al., 2017: 888), should be not a status issue but medically relevant because s/he is the specialist for the required treatment.^9^

The econimistic perception and reduction of the Civic OW *test* category to solely legal ‘equality’ seems to be the case for the formal setting of the German statutory Health system. The acceptance of the economic conception of the self and society theoretically would explain the state sanctioned market-organized private, first-class and public, second-class health insurance system. Analytically, the Industrial OW ‘technical efficiency’ moderates the drift towards more Market ‘competitiveness’. In absence of a Socialism OW as a convention of equivalence and the higher principal of ‘solidarity,’ the analytical means are missing in the OW framework for studying the composite OW arrangement: Instead, for the case of the German binary health system an analytical slippage (indicated by the arrows in Table 1) from the Civic to the Market OW via ideas of Industrial ‘efficiency’ surfaces.

**Table 1:** Categories of Civic, Industrial and Market OW and schematic OW drift in the German political economy of health

	Civic		Industrial		Market
Mode of evaluation (worth)	Collective interest ^a^	→	Technical efficiency	→	Price, cost
Test	Equality ^b^	→	Competence, reliability, planning	→	Market competitiveness
Form of relevant proof	Formal, official	→	Measurable: criteria, statistics	→	Monetary
Qualified objects	Rules and regulations, fundamental rights ^c^	→	Infrastructure, method, plan, project, technical object	→	Freely circulating market good or service
Qualified subjects	Citizens ^d^	→	Engineer, expert, professional	→	Customer, consumer, merchant, seller
Time formation	Perennial	→	Long-term planned future	→	Short-term, flexibility
Space formation	Detachment	→	Cartesian space	→	Globalization

^a^) The earlier term used by Boltanski and Thévenot (1999:368) is given, not the later term “collective welfare” (Thévenot et al., 2000: 241)

^b^) later amended to "equality and solidarity" in Thévenot et al. (2000: 241)

^c^) later amended to “rules and regulations, fundamental rights, welfare policies” in Thévenot et al. (2000: 241)

^d^) later amended to “equal citizens, solidarity unions" in Thévenot et al. (2000: 241)

Sources: Boltanski and Thévenot (1999: 368); Thévenot et al. (2000: 241); Modified by author

As will be shown in the following descriptive analysis, The economistic perception and reduction of the Civic OW *test* category to solely legal ‘equality’ results in ‘cost’ schemes applied to public ‘infrastructure’ (e.g., quality and cost-containment incentives for hospitals and drugs) and the definition of health ‘goods and services’ as commodities with a ‘price and cost’. The creation of a state-organized ‘formal and official’ (*form of relevant proof* for Civic OW) health Market – and not of a “quasi-market” (McMaster, 2002) – transforms originally ‘equal’ ‘citizens’ into un ‘equal’ ‘customers, consumers’. The situations of political economic infra*structure* that constructed and continuously construct the ‘unequal’ ‘citizen’ ‘customer’ are:

1. Situations of law-making: Elected government and members of parliament, who legally construct the binary health system in Germany,
2. Situations of law-applying: Minister and bureaucrats in the ministry of health, and ‘experts’ who plan the health ‘infrastructure’ and ‘cost’ schemes, creating an Industrialized market for health merchants (e.g., general practitioners, hospitals and care companies). These health ‘merchants and sellers’ regard patients as ‘customers’ of basic healthcare (when hospitalized due to an accident) and ‘consumers’ of preemptive health services (e.g., dental care).
3. Everyday interaction situations: Industrial ‘professionals,' such as doctors, nurses, and care workers, who treat patients not as ‘citizens’ who deserve ‘equal’ attention but as ‘customers and consumers’ who get first-class or second-class treatment.

### Brief Overview of the German Statutory Health System

Less OW abstract, the basic organization blocks of the German statutory health system are ‘monetary’ and employment status. For second-class, public health insurance, employees pay income dependent rates (on average 14.6% in 2020). Employees who earn more than € 60,750 before taxes per year, as well as self-employed persons (≈ 35%) can switch to first-class, private health insurance (PKV, 2017: 28). According to an OECD report (Colombo & Tapay, 2004), the opting out of the rich(er) part of society favoring health insurance Market means jeopardizing the German statutory health system. In more detail, Greß (2007: 29) summarizes the threats as follows:

Enrollees in private health insurance are healthier, have higher incomes and have fewer dependents than enrollees in [public] health insurance. Adverse selection decreases average premium income and at the same time increases average healthcare expenditures in social health insurance. As a consequence, financial sustainability of the public system declines. Moreover, financial incentives for healthcare providers have led to preferential treatment for privately insured patients in outpatient care.

In 2017, about 8.8 million people were privately insured (11% of the people insured in Germany) of whom about 50% were male, 32% female and 18% children (VDEK, 2019: 15). In 2011, the European Court (legal matter C-236/09) forced private health insurance companies to stop the discrimination of women by defining sex as a risk factor and to offer unisex rates. In addition to benefitting the rich(er), primarily male members of society, private health insurance provides wider health coverage and better patient treatment for lower costs. For example, according to an online portal of the association of private insurance companies, for basic health insurance employees pay minimum monthly rates of about € 90 (born in 2000), € 230 (born in 1980), and € 410 (born in 1960). Comfort and premium rates cost for fe*males born in 2000 about € 125 and € 260, in 1980 € 260 and € 395, and in 1960 € 535 and € 740. Of course, actual rates vary according to year of entry, co-payment and included ‘products and services’ (Market OW *objects*), and, for comfort and premium rates, pre-existent medical conditions. However, the portal emphasizes that the basic insurance might be “insufficient,”^10^ and that only the comfort and premium rates will guarantee the standard expected by privately insured ‘citizens’ (Table 2). The basic rate of private insurance covers more health ‘products and services’ than the basic public health insurance, for which an employee earning € 60,750 per year pays a monthly rate of about € 740.

**Table 2:** Private health insurance ‘products and services’ according to rates

	Basic	Comfort	Premium
*Outpatient*			
General treatment	+ +	+ +	+ +
Treatment by chief physician	+ +	+ +	+ +
Treatment by medical specialist	+	+	+ +
Drugs and bandages	+	+	+ +
Remedies (e.g., massages, speech therapy, ergo therapy and physiotherapy)	+	+	+ +
Adjuvants (e.g., glasses and contact lenses)	+	+	+ +
Healer/alternative practitioner	−	+	+ +
Psychotherapy	−	+	+ +
Alternative method of healing	−	+	+ +
*Inpatient*			
General hospital	+ +	+ +	+ +
Treatment	+ +	+ +	+ +
Single/double bedroom	−	+ +	+ +
Individual selection of doctor (chief physician)	−	+	+ +
*Dental*			
Teeth	+ +	+ +	+ +
Dentures (artificial denture, dental bridge and dental crown)	+	+	+
Inlays, dental implant and orthodontia	+	+	+

+  + No or minimal constraints (80%–100% reimbursements of costs)

+ Constraints (about 50%–80% reimbursements of costs)

− Not included

Source: https://www.krankenkassen.de/private-krankenversicherung/private-krankenversicherung-kosten/ (last accessed 04.10.2021); authors’ translation and arrangement

These privately insured ‘customers and consumers’ (*qualified subjects* of the Market OW) are treated better by health practitioners (the ‘merchants and sellers’) as private insurance pays higher prices for treatment and care, which led to a differentiation between the privately-insured first class and the publicly-insured second class:

An important difference affecting the supply of services is that for the same treatment, the compensation doctors receive for privately insured patients is, on average, 2.3 times as high as the compensation for publicly insured patients […]. Therefore, doctors have an incentive to treat privately insured patients first, and more intensely, possibly providing better treatment […]. For example, waiting times for privately insured patients are lower on average […]. (Hullegie & Klein, 2010: 1049)

The superior health treatment received by those who are already healthier and financially better-off increases their survival chances and quality of life (e.g., Kibele et al., 2016; Lungen et al., 2011). This non- ‘solidarity’ and in’equality’ is especially harmful for the socially less well-off, who are more likely to have to work in physically more demanding, chemically polluted and socially less-regarded jobs (Bor et al., 2017; Siegrist & Dragano, 2016). When these high-risk groups belong to the sickest and poorest, they benefit from extra state-sponsored targeting measures–in a way, a state compensation for legalized in’equality’ (Hinrichs, 2010).

The binary health system in Germany still displays an element of the 'equality' required by the Civic OW. Eventually, those once financially better-off can return to the public insurance system if their salary falls below a threshold, for example, after changing to a less well-paid or to a part-time job.^11^ In 2017, 133,000 individuals left the private insurance and returned to public insurance, while 129,300 individuals went the other way (PKV, 2017: 26). Trading rich(er) for poor(er) ‘citizens’ continuously increases financial pressure on the public health insurance system. In 2000, the German Parliament passed a law for to strictly limiting older people switching back from private to public health insurance.

### The German Industrialized Health Market

Previous German governments promoted this in’equality’ “because policy makers have concluded that mixed public–private coverage systems can better deliver desired health policy and social outcomes” (Colombo & Tapay, 2004: 16). As a result of a government efforts to achieve the desired health policy and social outcomes, German governments created legal boundaries for health “market regulation” (Boltanski & Thévenot, 2006: 266) by defining “legalistic-economic relations” (Boltanski & Thévenot, 2006: 239). Busse et al., (2017: 883) argue that “[s]ince the late 1990s, the German health system has moved towards integrated care and evidence-based healthcare, with new financial incentive schemes for both sickness funds and providers to improve quality and efficiency of care”. In this way, both the public and private healthcare systems orientates towards ‘price and cost,’ while ‘technical efficiency’ standard of the Industrial OW evaluates the health ‘infrastructure’ and medical ‘professionals’.^12^

For example, in the *Pharmaceutical Market Reform Act* of 2011, the German government reacted to the rising health ‘costs’ with “quality and cost-containment incentives” (Busse et al., 2017: 891) that required makers of “newly licensed pharmaceutical products [to] submit a dossier with sufficient data to assess the drug’s added benefit relative to existing products. […]. For drugs with an added benefit, the *Federal Association of Sickness Funds* would negotiate a reimbursement amount with the manufacturer”. The negotiation between the sickness funds (in German: *Krankenkassen*), comprising of 113 sickness funds organized in six associations, and pharmaceutical manufacturers creates a competitive Market (DEVK, 2019: 12). The result of health Market regulation are rapidly rinsing Civic ‘costs’. For example, the ‘cost’ containment for drugs has not been successful (they increased from € 29.4 billion in 2012 to € 37.7 billion in 2017; DEVK, 2019: 28). The *Hospital Structure Reform Act* of 2016, which tackles the rising costs of hospital treatment (increased from € 50.4 billion in 2007 to € 74.9 billion in 2017; DEVK, 2019: 28), offers no information concerning the quality issue, neither for drugs, nor for hospitals.

Accordingly, the current state of the OW framework only helps to *understand* the structural and construction dimensions resulting in the drift of public contributions creating private worth. However, the sociological analysis using the Civic OW of individuals’ obligation to have health insurance and the fact that in Germany the privately insured receive a better treatment than the publicly insured left an unsatisfactory question mark over what ‘equality’ means. First, the privately insured benefit more from medical services than the publicly insured. Second, additional private insurance (e.g., for dentures) further damages the ‘equality’ of the majority of individuals in Germany who are publicly insured by treating them according to the insurance Market ‘prices’ (*mode of evaluation* of the Market OW) they can afford. More and more publicly insured individuals buy partial private insurance for certain treatments that are no longer covered by public insurance, e.g., some forms of dental care (more than 25 million individuals in 2017; PKV, 2017: 32). In 2009, private additional health insurance covered 76.2% of the cost of dentures while public health insurance covered less than one quarter of the cost (23.8%) (VDEK, 2009: 20; Busse et al., 2017: 888).

The “sacrifice” or “economy” (Boltanski & Thévenot, 2006: 76) seems most beneficial for health ‘merchants,’ ‘sellers’ of pharmaceuticals and privately insured health ‘customers and consumers’ (*qualified subjects* of the Market OW), and least, or to a much lesser extent, beneficial for the vast majority of publicly insured German ‘citizens’ (*qualified subjects* in the Civic OW). Of course, individuals have to take care of their own health, but do so within the limits of genetic defects, socio-economic necessity (e.g., having to work in a polluted environment) and the effect of the global environment on human health, such as Ebola, SARS (Hanrieder, 2016), and recently the COVID-19 pandemics. Additionally, the rich(er) members of German society can afford to indulge in behavior that damages their health, while the majority is forced to use limited health ‘services’ and suffer additional limitations of individual freedom by (voluntarily) reporting to insurances sport activities or quitting smoking (Mathar & Jansen, 2015; Rosanvallon, 2014). However, unequal access to health ‘products and services’ is not justified or deserved by the general moral conduct of ‚citizens,’ but according to their differences in income and wealth.

### The ‘Equality’ of First-Class Market and Second-Class Industrial Health Treatment

The arrow-indicated drift in Table 1 analytically captures the construction dimension based on the current OW structural dimension. The Industrial organizing according to ‘technical efficiency’ and by considering ‘cost and prices’ (Market OW *mode of evaluation (worth)*) over health justify an OW compromise differentiating the population in Germany in in ‘equal’ first-class and second-class individuals according to annual income. In 2020, self-employed persons and employees who earn more than € 60,750 per year before taxes were entitled to private health insurance. In 2015, 43% of self-employed and 5% of employees were privately insured, and 5% of employees voluntarily remained in public health insurance.^13^ In spite of Germany being classified as an ‘equality’-oriented welfare state (e.g., Esping-Andersen, 1990; Zacher, 2013), 11% of the population is privately insured, 87% are publicly insured and “[t]he rest of the population (e.g., soldiers, police officers, and refugees) receives health insurance supported by specific governmental schemes” (Busse et al., 2017: 893), which also include civil servants (Scholz, 2018).

While refugees, poor and unemployed people are in need of welfare support, for example, Miller (1999) argues that police wo*men and soldiers in their potentially life-threatening service to the state could be entitled to deserve special (health insurance) treatment. However, what is the moral argument in the ‘collective interest’ for the rich(er) part of the German society deserving to be treated as being *more* ‘equal’ than the other ‘citizens’ concerning health insurance? It seems that the answers are a reload of the Bismarckian tradition of the binary health insurance system as a means to keep political stability and preventing social turmoil (Offe & Fuchs, 2007: 7; Zacher, 2013)–this time appeasing the rich(er) part of German society.

**Fig. 1:**
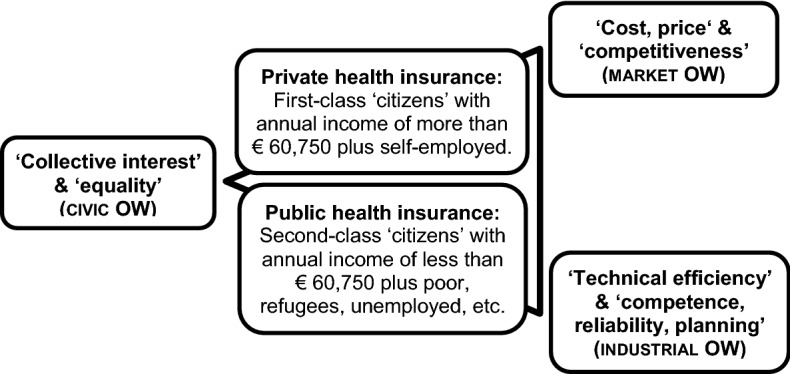
Triangle of in’equal’ first-class and second class ‘citizens’ in the German binary political economy of health according to *mode of evaluation (worth)* and *test* of the Civic, Industrial and Market OW

In Fig. 1, the triangular arrangement highlights the moral asymmetry ‘money’ (Market OW *form of relevant proof*) in form of annual income defining first- and second-class ‘citizens’ as ‘technically efficient’ compromise in the ‘collective interest’. The arrowhead arena of Fig. 1 emphasizes the analyzed constructive drift in the German binary political economy of health, i.e., of Market and of Industrial conventions driving the Civic construction of the German statutory health system–explaining the social distribution of wealth and health. However, with the current analytical blank spot, the plural OW framework does only allow this particular analysis. In the following section, I discuss systematically the extension of the OW framework by considering the basic social human issue of Socialism.

## Including the Socialism OW in the OW Framework

### The Seventh Axiom: Analytical Adequacy

For the construction of the OW framework, Boltanski and Thévenot (2006) defined six axioms (common humanity (a1), differentiation (a2), common dignity (a3), order of worth (a4), investment formula (a5), and common good (a6); Table 5). While the latter two construct government modes of a political economy, a3 and a4 define states or ways of human order that have to comply with the principles of differentiation and of common humanity. Considering a1 (common humanity) and a2 (differentiation), the construction of a statutory binary health system in Germany raises the question whether it accomplishes the common humanity axiom or not (e.g., normative exclusion of treating humans as subhumans; Boltanski & Thévenot, 2006: 74). Of course, not all humans are German ‘citizens’. However, as humans, publicly insured refugees are treated equivalent to second-class ‘citizens’. Due to the difference between first-class and second-class ‘citizens,’ in the ‘collective interest,’ not all ‘citizens’ are regarded according to the principle of equivalence of ‘equality’ (Boltanski & Thévenot, 2006: 259f., 266). For the German case, the OW framework neither provides a tool for the explanation of treating refugees as second-class health insured ‘citizens,’ nor the ‘equality’ exceeding human selfishness of private health insurance captured by the Market OW.

The systematic solution to such an empirical problem is the suggestion of a seventh axiom of analytical adequacy (a7). This axiom is not relevant for the construction of a specific OW but addresses the structural dimension of the OW framework. This axiom is of double use. First, the analytical adequacy axiom demands researchers to use as many OW as applicable for the analysis of a social situation, i.e., the study of the construction and interaction dimensions (see also Boltanski & Thévenot, 2006: 37f.). Second, the analytical adequacy axiom can help to empirically and/or theoretically (Boltanski, 2012: 98f.) identify an analytical blank-spot in the OW framework for sociological analysis.

As shown above, the methodic-theoretical blank-spot identified in the OW framework is ‘solidarity’-based ‘(collective) welfare’ (Socialism OW *mode of evaluation (worth)*), which relates to the socio-biological motivation to survive. Originally, private health insurance in what is now Germany dates back to the Middle Ages, when craft and trade guilds organized ‘solidarity’ funds for their members (Bärnighausen & Sauerborn, 2002). Various authors (e.g., Busse et al., 2017; Hinrichs, 2010) make it very clear that this health insurance rooted in the ‘solidarity’ principle held between equal members of the guilds. Accordingly, the individual and economic survival chances of craft and guild members stemmed from ‘solidarity’ among equals bound by “instrumental associations” (Miller, 1999: 27), which cater for their members healthy and functioning organism to survive in medieval times. This Industrial OW organizing of ‘equal’ guild members would be a compromise, as described by Boltanski and Thévenot (2006). In comparison to the compromising Industrial ‘solidarity,’ the difference is an indifferent general or higher principle of ‘solidarity’ as *test*-category for a (Socialism) OW.

**Fig. 2:**
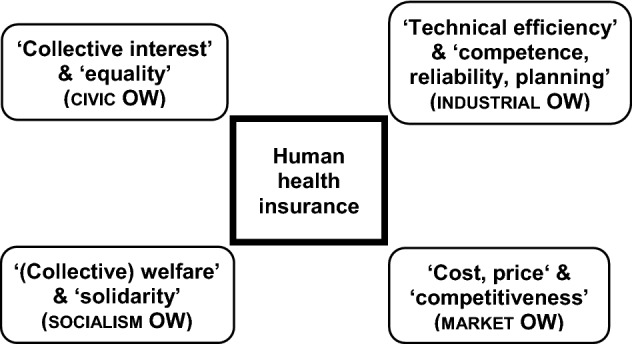
Differentiated analysis of a political economy of health according to *mode of evaluation (worth)* and *test* categories of the Civic, Industrial, Market, and Socialism OW

Other members of the human race, such as aristocrats, local gentry, helots, etc., living in the area had no health insurance, and had to rely on Domestic OW structures defining ‘solidarity’ interaction in case of family members falling ill (Boltanski & Thévenot, 2006: 90–98). The German subsidiarity legal tradition (Taylor, 2006) preserves such Domestic OW elements, for example, in the case of taking care of sick elderly family members, like in the ‘customary past’ (*time formation*). Nevertheless, such Domestic OW elements are minor *subjects* to the *qualified objects* of ‘rules and regulations, fundamental rights’ (Civic OW), health care ‘infrastructure, methods, and plan’ (Industrial OW), and elderly care at home either by family members or hired personnel as ‘freely circulating market good or service’ (Market OW) covered (partly) by (public) health insurance. While the Domestic OW is of minor relevance, the remaining OW (e.g., Fame and Inspired) are irrelevant for the general analysis of human health insurance. Accordingly, as expressed by the second part of the analytical adequacy axiom (a7), for sociologically analyzing human health insurance the Socialism OW has to be added to the conventional economic approach focusing only on Civic, Industrial and Market OW organizing of communality (Fig. 2).

On the trajectory from empirical reflection towards the methodic-theoretical construction of OW, the following sections will summarize and further elaborate the Socialism OW according to the abstract categories of the OW framework. The overview of both additional OW is then complemented by a systematic justification of the canonical text and axiom application to the Socialism OW.

### Characteristics of the Socialism OW

The word ‘socialism’ finds its root in the Latin *sociare*, which means to combine or to share. The related, more technical term in Roman and then medieval law was *societas*. This latter word could mean companionship and fellowship as well as the more legalistic idea of a consensual contract between free men. (Vincent, 2010: 83)

For Rosanvallon (2013: 286) a community is composed of a group of individuals who are bound to each other by reciprocity and are braided together by a dense web of hopes and hardships. Accordingly, the singularity of an individual is constituted by diversity, the difference in relation to other singularities (Rosanvallon, 2013: 260). The *qualified subjects* of the Socialism OW (Table 3) are ‘solidaristic singularities,’^14^ for whom ‘equality’ means a communal, democratic and reciprocal recognition of each other’s diversity. As emphasized in the quote by Vincent (2010: 83), such a non-formal, however, consensually binding idea between humans as ‘solidaristic singularities’ aims for a polity, where affirmative action and positive discrimination according to ethnicity, gender, skin color, etc. are superfluous. The formation of ‘solidaristic singularities’ depends on the subsidiarity principle, i.e., the local polity is responsible for fostering communality and reciprocity. As communality is a feature shared by all OW, by adding it in brackets to the *test* category, ‘(communal and reciprocal) solidarity’ become explicit analytical reference categories in qualifying ‘solidarity’. Accordingly, ‘solidarity’ is not a technical issue but requires a communal and reciprocal conscience as explained earlier by Simmel (1992/1908) in *Sociology: Inquiries into the Construction of Social Forms*.

**Table 3:** Categorized abstractions of the Socialism OW

	Socialism OW
Mode of evaluation (worth)	(Collective) welfare
Test	(Communal and reciprocal) solidarity
Form of relevant proof	Safety, quality of life
Qualified objects	Health, accident, pension, subsistence, unemployment
Qualified subjects	Solidaristic singularities
Time formation	Human lifetime
Space formation	Nation state, alliance

The organization of the nation state is supposed to distribute unconditional social support and services by providing ‘rules and regulations’ (*qualified objects* of the Civic OW). In Rosanvallon’s (2013: 268) polito-philosophical utopia, the distributive ‘rules and regulations’ have to meet two ethical dimensions: first, they generate equitable laws or “just rules” (generality pole) and second, they agree with singularities’ benefits with respect to “forms of attentive behavior” (particularity pole). Based on the general assumption that absolute ‘equality’ of all will not be achievable, Rosanvallon (2013) stresses the necessity of a legal definition of equal rights and duties to assure ‘solidarity’. However, ‘solidarity’ among singularities depends greatly on the legitimacy of redistribution by the state, which has to establish trust in singularities that the rich(er) pay their taxes, while the middle class is not disregarded and the poor do not take advantage of social benefits (Rosanvallon, 2013: 296). Rosanvallon (2013: 277) complements the condition of reciprocity with the condition of communality–based on the understanding of the Latin *civis* (companions with a shared place of residence in *societas*). Shared and legally defined rights and duties for singularities, which construct a social space for singularities, also construct communality.

In Table 3, the *form of relevant proof* is defined as’quality of life, safety,’ which are individual needs that give social stability. For the Socialism OW and its *mode of evaluation (worth)*, ‘(collective) welfare’–the normative utopia of a higher common good (Table 4) –, the *test* of ‘solidarity’ is the principle “tacitly enclosed in the arrangement of ordinary situations or set in tension in compromises” (Boltanski, 2012: 98f.). The term ‘(collective) welfare’ explicitly emphasizes the doing well of the whole community–an idea developed among utopian socialist (e.g., Saint-Simon; Vincent, 2010: 84, 90) –, and is distinctly different from the limited ‘solidarity’ expressed by the political economy of capitalist welfare states (e.g., Batifoulier et al., 2019, 2021; Chiapello & Knoll, 2020; Esping-Anderson, 1990). The “world of objects” (Boltanski, 2012: 99) or the *qualified objects* of the Socialism OW are defined in Table 3 as ‘accident, health, pension, subsistence and unemployment,’ based on Briggs’ (1961) ideal benefits for doing well in Western societies. ‘Accident’ refers to both occupational and private accidents that keep individuals from earning their living and taking care of themselves. ‘Unemployment’ and ‘pension’ are both related to work in the definition of social rights, e.g., concerning poverty of women in old age (Anderson, 2019). The question of ‘solidarity’ is just as relevant for what belongs to ‘subsistence’. On the one hand, ‘subsistence’ summarizes basic human needs such as food, water, shelter, etc. On the other hand, ‘subsistence’ includes objects such as electricity, technical artifacts, etc., which are considered necessary for a socially inclusive way of life in Germany and elsewhere (Obinger & Petersen, 2019; Zacher, 2013).

## Selection of the Canonical Text according to OW Axioms

### Justification of Canonical Text by Rosanvallon

The previous section explained the main categories and definitions of the Socialism OW. While trying to avoid redundancy as much as possible, this and the following section explain and justify the selection of canonical texts and OW axioms. Boltanski and Thévenot (2006) define five criteria for the selection of a canonical text (Table 4) and six axioms, which are based on constructions of political philosophy and give “direction to the ordinary sense of what is just” (Boltanski & Thévenot, 2006: 74). Boltanski and Thévenot (2006) justify the selection of a canonical text based on the content of the text, especially whether it has a systematically presented key concept. Accordingly, a selected canonical text does not have to be the best text in comparison to other political philosophies, which was questioned by Honneth (2010). Honneth (2010: 387) further criticized that “Boltanski and Thévenot must not pretend that there are six equally available models of justice for all spheres of coordination among individual actions”. Thévenot responded in an interview with Blokker and Brighenti (2011: 392) that Honneth (2010) was misled “to take the book as a theory of hierarchical structures of social status based on individual desert” instead of an abstract, context-transcendent and plural framework for legitimate critique and justice in and of social situations.

The canonical text selected when constructing the Socialism OW (Rosanvallon, 2013) is presented in Table 4 by focusing on the analytical content and without elaborate explanations. Based on Table 3 and using the same rationales, Table 5 presents the Socialism OW axioms, also in a relatively condensed form. In spite of the clear methodic-theoretical indication of the content of the six axioms, the axioms are not made explicit in the presentation of the Civic, Domestic, Fame, Industrial, Inspired and Market OW by Boltanski and Thévenot (2006: 74–124). Accordingly, the tabular presentation of the Socialism OW should be treated as a step toward the transparent and methodic-theoretical presentation of OW construction, in support of the analytical adequacy axiom (a7).

**Table 4:** Five criteria for selecting a canonical text by Boltanski and Thévenot (2006: 71–74), applied to Rosanvallon (2013)

Criteria	Socialism OW
(a) The selected text should be (one of) the earliest political text(s) to present the polity in a systematic form. The grammar of the political text should provide for general formulations, i.e., be applicable to everyone and in all situations, which validate the operating customs, procedures, rules and settlements on the local level. The higher common principle must be satisfied “in order to sustain *justifications*” (Boltanski & Thévenot, 2006: 66). However, not all aspects of a canonical text are relevant. For example, the Civic OW defines the “State” (Boltanski & Thévenot, 2006: 72) based on a (legal) equality of citizens	Rosanvallon’s *Society of Equals* is selected as a canonical text because he is (among) the first to focus on the ‘singularity’ (individual) as a ‘solidaristic human being’ that constructs a communal and reciprocal polity (social world). The ‘solidarity’ among singularities is rooted in the esteem for oneself and others (see also Honneth, 2005: 121). Based on a historical and contemporary analysis of the French and US societies, Rosanvallon theorizes (Chap. 5) that the sociality of individual human beings can be singular as well as communal and reciprocal. In contrast to other accounts of alienation in the individualized, singular world (e.g., Bauman, 2000; Reckwitz, 2020), Rosanvallon provides a clear grammar to describe the communal and reciprocal ‘solidarity’ between singularities (see also Rosanvallon, 2014)
(b) The text needs to define a higher common principle, which is used in a socially structured situation for the construction of worth, [and to] present “a form of sacrifice and a form of common good possessing universal validity” (Boltanski & Thévenot, 2006: 72)	The higher common principle is communal and reciprocal ‘solidarity’ between singularities. Worth is based on the ethical continuum of singularities’ ‘solidarity’: the generality pole consists of “just rules,” and the particularity pole consists of agreement on singularities’ benefits being tied to “forms of attentive behavior” (Rosanvallon, 2013: 268). The worth of the higher common principle and its expression of subsidiarity ideally reduce the need for legal ‘rules and regulations’ (Civic OW) to a minimum. However, the sacrifice of singularities’ communal and reciprocal solidarity threatens social stability
(c) The text has to be explicitly political in the way the author argues for the “principles of justice that govern the polity” (Boltanski & Thévenot, 2006: 72)	The ‘solidarity’ of singularities, based on mutual esteem, is fundamental for the social construction of a communal and reciprocal polity
(d) The canonical text has to aim to establish practical trust within a polity by constructing a “natural order so as to institute situations that are stabilized by recourse to a higher common principle” (Boltanski & Thévenot, 2006: 73)	Rosanvallon argues in Chapter 4 that the natural trust known in industrial modernity (e.g., cultural conformity, rationalization, technicalization and expansion of the welfare state) is no longer valued in late modernity. In Chapter 5, the idea of singularities’ communal and reciprocal solidarity might be judged utopian in 2022. However, the Socialism “world is possible – that is, logically possible, cohesive and robust” (Boltanski, 2012: 99) based on Rosanvallon’s (2013) political philosophy of the *Society of Equals*
(e) This criterion is ambiguous. Boltanski and Thévenot (2006: 74) start by postulating that the text should be “widely known,” and then specify that the text's use to formulate political technologies is a more important element of this criterion. Political technologies are defined as the “construction of instruments for establishing equivalence that are of highly general validity or for the justification of such instruments” (Boltanski & Thévenot, 2006: 74)	Of course, in comparison to Rousseau’s *Social Contract*, which is used by Boltanski and Thévenot (2006) to justify ‘legal’ structures (Civic OW), the work by Rosanvallon (2013) was published only recently. Rosanvallon (2013) systematically analyzes from a historical perspective the American and French dissonance of ideals in the evolution of non-solidaristic social structures of singularities life chances. In particular, Rosanvallon (2013) describes the political technologies of devaluation of singularities by the five different compensation types of social justice Rosanvallon (2013) defines the arrangements and arguments for esteem-based ‘solidarity’ as the core of a polity of communal and reciprocal singularities. Accordingly, ‘solidarity’ is the *test* for the justification of worth of the Socialism OW

### OW Axioms Applied to Socialism OW

The axioms (Table 5) used to construct an OW consist of two principles (common humanity and differentiation), two ways of human order (common dignity and order of worth) and two government modes of a political economy (investment formula and common good). According to Boltanski and Thévenot (2006: 77), “the definition of the common good is the keystone of the construction that has to ensure the compatibility between” the antagonistic requirements of a shared common humanity based on a common identity of all singularities in a polity and “the requirements of order governing this humanity”.

**Table 5:** Six Axioms by Boltanski and Thévenot (2006: 74–77), applied to the Socialism OW

Axioms	Socialism OW
Principle of common humanity [a1]	
Members of a polity who are capable of identifying with and reaching an agreement about a common definition of humanity (presupposition excludes slaves and subhumans)	‘Common welfare’ is based on the communal and reciprocal ‘solidaristic’ assurance of safety and quality of life when there is contingent loss of health (e.g., due to an accident), subsistence (e.g., food) and work (e.g., retirement)
Principle of differentiation [a2]	
Polity members have at least two possible *states* of existence that preserve personal particularities and may have as many states as the number of existing people	The possible states are situated on a continuum between ‘solidarity’ and indifference. According to Rosanvallon (2013: 260), the singularity of an individual is constituted by diversity, the difference in relation to other singularities. The diversity of ‘solidaristic singularities’ supports ‘equality,’ which is based on a democratic and reciprocal recognition of the other
Common dignity [a3]	
All members of a polity have potentially identical power to access all the different states of the multistate humanity	A common welfare system consists of singularities who provide for the means of welfare support and those who benefit from it. Ideally, singularities first provide for and then benefit from welfare (e.g., work and unemployment benefits). However, access to the different states in the common welfare system might be limited due to unfortunate circumstances (e.g., an accident)
Order of worth [a4]	
Compromises, disputes, disagreements and justification are necessary to achieve a ranking of polities that express a range of values (e.g., for the common good)	Need is the worth creating the order of ‘common welfare’. Communal and reciprocal singularities are morally required to balance their personal needs with those of others through ‘solidarity’. According to the subsidiarity principle, the State is supposed to define legal procedures to prevent fraud by members of the polity who are either non- ‘solidaristic’ or overly dependent
Investment formula [a5]	
Human beings with equal power to access all states (when a higher state equates to a greater degree of happiness) have to balance the benefits against the costs or sacrifices made to access higher and lower states	The investment formula of singularities in the higher states is based on the calculation that it could be me in need of ‘common welfare’ sometime in the unknown future. The communal investment formula is based on the political economy of a singularities’ potentially not being able to provide for welfare needs. The investment formula of the lower states is based on a singularities’ potential to avoid common welfare as far as possible
Common good [a6]	
This states that a good or happiness correlates to the higher or lower rank of a state and is not beneficial in a similar way to all members of a polity	The paradox of solidaristic common ‘welfare’ is, in the highest state, the willingness to contribute to it and at the same time hope not to be in need of it. In contrast to this highest state, the lowest state is defined by the paradox of being unwilling to contribute but knowing that common ‘welfare’ is a safety net to rescue a singularity in need

## Conclusion

This paper argued for the addition of the Socialism OW and analytical adequacy axiom to the great research program of the OW framework. The qualified empirical case study of the German statutory health system shows the applicability of the OW framework beyond France. Accordingly, the analytical OW framework and its abstract, methodic-theoretical categories, can be considered context-transcendent tools for the context-dependent social analysis in and comparative analysis of Western democracies. In addition, the Civic OW problem discovery and descriptive analysis of the German binary health system provides ample proof for the applicability of the OW framework for analytically understanding how plural OW operate within larger systems as coordinative devices of investments in forms on the meso- and macro-levels. Accordingly, the potential of the OW framework is shown to encompass the systematic analysis of.

1. Social processes as situations of justification and critique in a situation (e.g., empirically analyzing specific processes of coordination–that is, *tests* and other category-led OW expressions as well as by deductively applying OW related grammars),
2. A situation of (temporary) agreements, for example, of investments in forms.

This article shows that the methodological clarity of the research program (Boltanski, 2012; Boltanski & Thévenot, 2006) is an invitation for the application, (experimental) *test*ing and further empirical development of the OW research program.

## Footnotes

1 Diaz-Bone (2011: 44) emphasizes the systematic, holistic, analytic dimension of the term methodic in contrast to the *logos* of rational, methodological reasoning.

2 To facilitate reading, the OW are identified in the text using as proper noun (startig with a capital letter), the categories are italicized and identification terms are given in single quotation marks.

3 For example, Brunkhorst (2005) shows in a genealogy the heterogeneity of comprehending ‘solidarity’ in various contexts.

4 I want to thank Philippe Batifoulier for pointing this out.

5 The coarse, first-class and second-class differentiation suffices for the argument of this article. Actually, private insurances for additional health ‘goods and services’ (Market OW *objects*), such as dentures and treatments by individually chosen chief physicians (Busse et al., 2017: 888), does in fact add many more sub-divisions of individual’s health insurance classes.

6 According to Walton (1993) and others (Mazzucato, 2019; Piketty, 2021), the analysis of a political economy addresses the distribution of social resources within a society or a nation state and investigates its mechanisms and institutions, for example, human wealth and health (Batifoulier et al., 2019; Palier, 2010).

7 Concerning the fourth spirit of capitalism, the Socialism OW creates a link to critical efforts for overcoming the current economic system based on the capitalist ideology (Piketty, 2020). For Piketty (2021: 2) is socialism “the most appropriate term to describe the idea of an alternative economic system to capitalism”. As alternative to the present hypercapitalism, the “new form of socialism” is “participative and decentralized, federal and democratic, ecological, multiracial, and feminist” (Piketty, 2021: 2).

8 Alexander (1997) is the reference in the endnote by Thévenot et al., (2000: 269).

9 The absence of ‘solidarity’ and, as a consequence, of ‘equality’ in health insurance is not only a German phenomenon. For example, Rosanvallon (2014) predicted that the declining ‘solidarity’ among singularities would mean the end of French insurance. Rosanvallon (2014) based his prediction on the historical, anthropological and sociological analyses of French and US societies, in which the anti-democratic evolution of the singularities and the erosion of ‘solidarity,’ communality and reciprocity between singularities destroy those societies that originally aimed for Civic ‘equality’. In contrast to the French welfare state (Nielsen, 2018; Palier, 2010), and in spite of being classified as a liberal welfare system by Esping-Andersen (1990), the political economy of US healthcare is not redistributive. In the US, health ‘goods and services’ (*qualified objects* in the market OW) are offered primarily to the employed–but only during employment–and to those who can afford it (Bor et al., 2017; Dickman et al., 2017; Gaffney & McCormick, 2017).

10 https://www.krankenkassen.de/private-krankenversicherung/private-krankenversicherung-kosten/ (last accessed 04.10.2021).

11 Source: https://www.krankenversicherung.net/rueckkehr-gesetzliche (last accessed: 04.10.2021).

12 The ‘technical efficiency’ of public health insurance is better: the administration cost of public health insurance is 4.9% and of private health insurance is 8.5% in 2017 (VDEK, 2019: 15). Unfortunately, no information is provided on service quality and satisfaction of those on public vs. private insurance.

13 See: https://www.destatis.de/DE/Themen/Arbeit/Arbeitsmarkt/Qualitaet-Arbeit/Dimension-2/krankenversicherungsschutz.html (last accessed: 01.04.2022).

14 The term’solidaristic singularities’ is inspired by Miller (1999: 26), who philosophically addresses ‘solidaristic communities’ as a social or interaction sphere. For Miller (1999: 26, 38) ‘solidarity’ is part of a community sphere categorizing individuals with a common identity or common culture, kinship, acquaintance, and nationality.
